# Leishmania Induces Survival, Proliferation and Elevated Cellular dNTP Levels in Human Monocytes Promoting Acceleration of HIV Co-Infection

**DOI:** 10.1371/journal.ppat.1002635

**Published:** 2012-04-05

**Authors:** David J. Mock, Joseph A. Hollenbaugh, Waaqo Daddacha, Michael G. Overstreet, Chris A. Lazarski, Deborah J. Fowell, Baek Kim

**Affiliations:** 1 Department of Biomolecular Genetics, University of Rochester Medical Center, Rochester, New York, United States of America; 2 Department of Microbiology and Immunology, University of Rochester Medical Center, Rochester, New York, United States of America; 3 Center of Vaccine Biology and Immunology, University of Rochester Medical Center, Rochester, New York, United States of America; 4 GenVec, Inc., Gaithersburg, Maryland, United States of America; Fred Hutchinson Cancer Research Center, United States of America

## Abstract

Leishmaniasis is a parasitic disease that is widely prevalent in many tropical and sub-tropical regions of the world. Infection with *Leishmania* has been recognized to induce a striking acceleration of Human Immunodeficiency Virus Type 1 (HIV-1) infection in coinfected individuals through as yet incompletely understood mechanisms. Cells of the monocyte/macrophage lineage are the predominant cell types coinfected by both pathogens. Monocytes and macrophages contain extremely low levels of deoxynucleoside triphosphates (dNTPs) due to their lack of cell cycling and S phase, where dNTP biosynthesis is specifically activated. Lentiviruses, such as HIV-1, are unique among retroviruses in their ability to replicate in these non-dividing cells due, at least in part, to their highly efficient reverse transcriptase (RT). Nonetheless, viral replication progresses more efficiently in the setting of higher intracellular dNTP concentrations related to enhanced enzyme kinetics of the viral RT. In the present study, *in vitro* infection of CD14+ peripheral blood-derived human monocytes with *Leishmania major* was found to induce differentiation, marked elevation of cellular p53R2 ribonucleotide reductase subunit and R2 subunit expression. The R2 subunit is restricted to the S phase of the cell cycle. Our dNTP assay demonstrated significant elevation of intracellular monocyte-derived macrophages (MDMs) dNTP concentrations in *Leishmania*-infected cell populations as compared to control cells. Infection of *Leishmania*-maturated MDMs with a pseudotyped GFP expressing HIV-1 resulted in increased numbers of GFP+ cells in the *Leishmania*-maturated MDMs as compared to control cells. Interestingly, a sub-population of *Leishmania*-maturated MDMs was found to have re-entered the cell cycle, as demonstrated by BrdU labeling. In conclusion, *Leishmania* infection of primary human monocytes promotes the induction of an S phase environment and elevated dNTP levels with notable elevation of HIV-1 expression in the setting of coinfection.

## Author Summary

Leishmaniasis is a parasitic disease that infects several human host immune cells, including neutrophils, monocytes, and macrophages. Moreover, while HIV-1 infects monocytes and macrophages, only the infected macrophages productively release viral progenies. Importantly, patients coinfected with both pathogens progress more rapidly to AIDS. In this study, we examine how *Leishmania major* changes the cellular environment of monocytes *in vitro*. We found that *Leishmania*-infected monocytes actively mature into macrophages in the absence of GM-CSF, and that these cells up-regulate the expression of ribonucleotide reductase, an enzyme that catalyzes the formation of deoxynucleoside triphosphates (dNTPs). We confirmed the elevation of dNTP concentrations using a very sensitive dNTP assay for monocytes and monocyte-maturated macrophages. Collectively, these data support a model in which infection of monocytes with *Leishmania* elevates the intracellular dNTP pools, which is one of the natural anti-viral blocks to HIV-1 infection in monocytes and macrophages in patients.

## Introduction

Leishmaniasis has recently been recognized to be both one of the world's most neglected and most important parasitic diseases, threatening an estimated 350 million people worldwide [1], [2]. Surveys have estimated that approximately 12 million people are currently infected with 2 million new cases reported yearly, primarily afflicting the world's poorest populations in some 88 countries [3]. Leishmaniasis is transmitted to humans by the bite of the female Phlebotomine sandfly upon taking a blood meal [4]. Infection results in three basic clinical presentations. Cutaneous and mucocutaneous leishmaniasis are disfiguring and even mutilating diseases, while visceral leishmaniasis (VL) is characterized by fever, massive hepatosplenomegaly, pancytopenia, and a wasting syndrome called Kala-azar, which is nearly uniformly fatal without treatment [5], [6].

Early after the emergence of the global Human Immunodeficiency Virus Type 1 (HIV-1) epidemic, clinicians recognized that reciprocal activation of each pathogen by the other frequently occurred. It was noted, on the one hand, that infection with HIV-1 modifies the natural history of leishmaniasis, leading to 100–2,230 times increase in the risk of developing VL and reducing the likelihood of a therapeutic response [7]–[11]. At the same time, VL was shown to induce activation of latent HIV-1, increase viral load, and cause a striking acceleration in the progression of asymptomatic HIV-1 infection to AIDS that corresponded to a reduction of life expectancy in patients [12]–[15]. Similarly, it was recognized that monocytes and macrophages are the primary cell types coinfected with both HIV-1 and *Leishmania*. Initial studies demonstrated that *Leishmania* coinfection reactivated HIV-1 replication in latently infected monocytoid cell lines [16]. Subsequent studies in primary MDMs coinfected with *L. infantum* and HIV-1 also found enhanced HIV-1 replication associated with increased secretion of the pro-inflammatory cytokines TNF-α, IL-1α, and IL-6. In these experiments, HIV-1 replication, as measured by p24 ELISA, was reduced in the presence of either chemical inhibitors or blocking antibodies to these three cytokines [17].

Human monocytes circulate in the blood and reside in bone marrow and spleen and are generally believed not to proliferate in the steady state [18], [19]. However there is an emerging awareness that human monocytes possess far greater heterogeneity than originally perceived, and subpopulations of monocytes have recently been described that can re-enter the cell cycle in response to both Macrophage- and Granulocyte Macrophage-Colony Stimulating Factors (M-CSF and GM-CSF, respectively) [20]–[22]. Proliferation of these presumably immature peripheral blood monocyte subpopulations has been demonstrated by multiple techniques including uptake of 5-bromo-2′-deoxyuridine (BrdU) and CFSE labeling, leading to this population being termed “proliferative monocytes” [23], [24].

Such cellular proliferative capacity has important implications because cellular dNTP levels correlate directly with the replicative capacity of mammalian cells [25]. Consistent with this observation, a variety of studies, including those from our laboratory, have reported that dNTP levels are consistently higher in dividing versus non-dividing cells [25]–[31]. Among the retroviruses, HIV-1 possesses the unique ability to infect both dividing (activated CD4+ T cells) and non-dividing cells (macrophages). This ability is due, at least in part, to the evolutionary adaptation of its reverse transcriptase (RT) to function under conditions of extremely limited dNTP availability [32]. However, as noted for the replicative capacity of mammalian cells, HIV-1 replication efficiency is also directly correlated with cellular dNTP concentrations and proceeds with far greater efficiency in both tumor cells and PHA-stimulated CD4+ T cells, in which the average dNTP levels are 150–225 times higher than that of non-dividing MDMs [32], [33]. Several recent studies have shown that HIV-2 Vpx protein promotes the degradation of the SAMHD1, a host anti-viral restriction factor [34]–[37]. Recently, SAMHD1 was shown to function as a dNTP hydrolase [38], [39], limiting the cellular dNTP pool and restricting HIV-1 replication in cells of myeloid lineage [40]. Moreover, our recent paper shows a direct connection between SAMHD1 degradation, an increase in dNTP levels and enhanced transduction of HIV-1 in myeloid cells [41].

In the present study, we found that *in vitro* infection of freshly isolated, undifferentiated CD14+ primary human monocytes with *Leishmania* consistently led to maturation into macrophages and to higher cell numbers over time as compared to uninfected control cells. In addition to the inhibition of apoptosis previously reported in *Leishmania*-infected MDMs, we also report the unexpected finding that a sub-population of CD14+ human MDMs proliferate in response to *Leishmania*, as measured by BrdU incorporation at days 12–14 after infection. As the efficiency of HIV-1 RT DNA synthesis and subsequent viral replication are directly dependent on cellular dNTP concentration, we subsequently employed a highly sensitive single nucleotide incorporation assay that was recently developed in our laboratory to measure cellular dNTP concentration [32], [42], [43]. We found a marked increase in the content of dNTPs in *Leishmania*-maturated MDMs as compared to uninfected control cells. Consistent with this observation, elevated levels of ribonucleotide reductase (RNR), the rate-limiting enzyme for dNTP synthesis, was also found in *Leishmania*-maturated MDMs as compared to control cells. Finally, we found significantly enhanced expression and transcription of a GFP-expressing pseudotyped HIV-1 (HIV-1 D3 GFP) in *Leishmania*-maturated MDMs as compared to control cultures as assayed by FACS analysis of HIV-1 D3 GFP expressing cells and qPCR for 2 LTR-circle copy number.

As noted above, previous studies have suggested a role for *Leishmania* infection of monocytes causing the induction of pro-inflammatory cytokines as a stimulus to HIV-1 replication in coinfected cells. Our data support a novel model whereby *Leishmania* infection stimulates monocytes' differentiation and cell division. Consistent with the increased proliferation capacity, *Leishmania* infection increases cellular dNTP concentrations that facilitate enhanced HIV-1 coinfection.

## Results

### Kinetic analysis of cell survival

The effect of *Leishmania* infection on cell survival of primary human monocytes was examined over a time course of 28 days from eleven individual donors. Preliminary experiments were performed to examine potential effects of both heat-inactivated *Leishmania* (also applied to monocytes at an MOI = 7) and day 7 conditioned medium from *Leishmania*-infected monocytes re-applied to freshly isolated monocytes. These experiments demonstrated no significant effects on monocyte cell survival, maturation, or proliferation (data not shown). In parallel experiments, *Leishmania* labeled with the vital dye PKH showed that at an MOI = 7 virtually all monocytes within the culture became infected (Figure S1). This MOI is well within the range of those previously published [16], [17].

**Figure 1:**
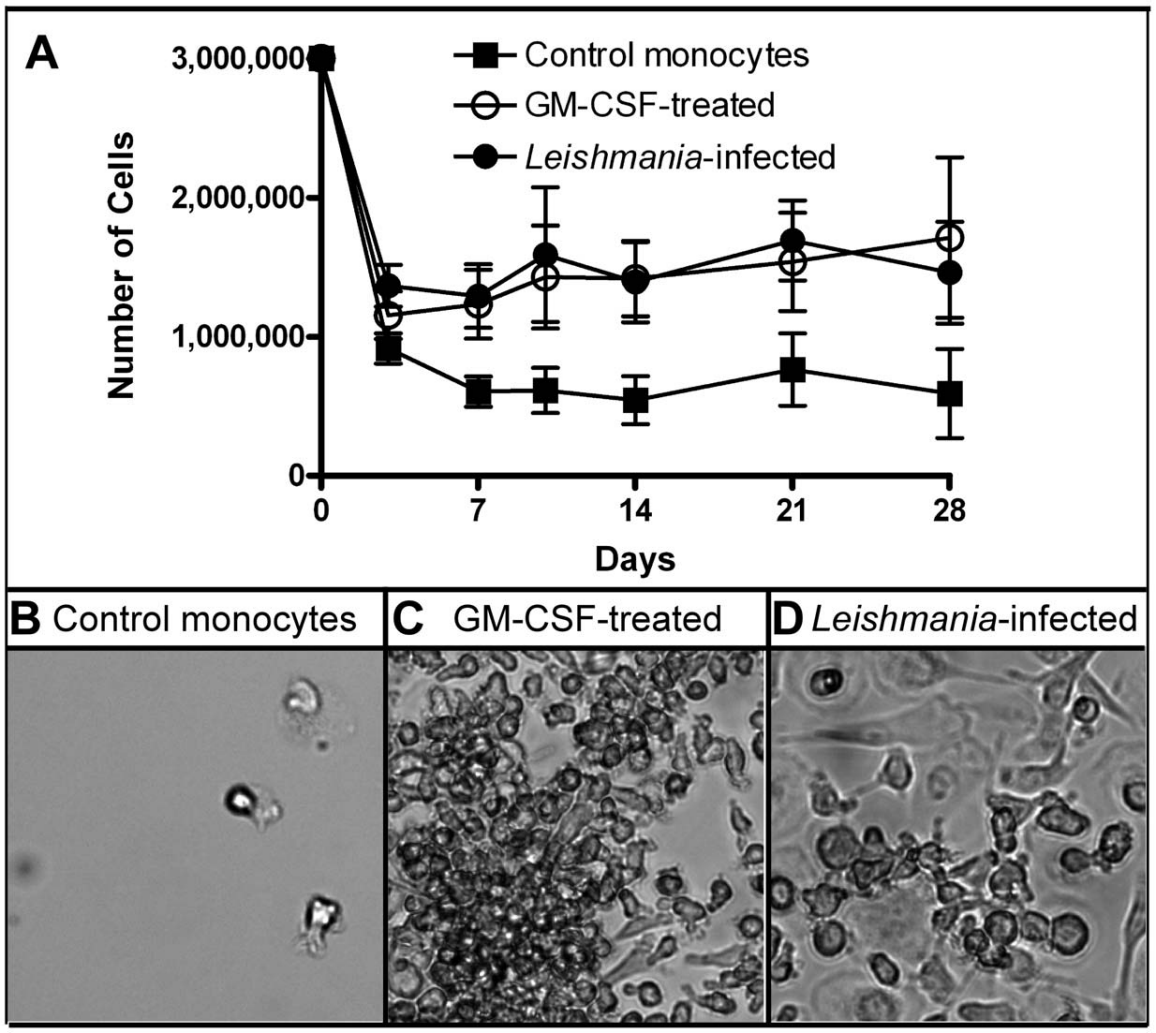
Determining cell viability. A) 1×10^6^ monocytes/well were plated and then left untreated (control), GM-CSF-treated or *Leishmania*-infected at an MOI = 7. At various days afterwards, three wells/condition were collected, pooled and the cell numbers determined. Nine independent donors were examined. B–D) Images were captured using bright field microscope. Control cells had very few adherent cells as compared to GM-CSF-treated and *Leishmania*-infected cell cultures.

Purified human monocytes were cultured at 1×10^6^ cells/well in 6 well dishes, and three wells from each of three culture conditions were combined and counted: 1) RPMI media with 10% FBS (“control cells”), 2) RPMI media with 10% FBS plus 5 ng/ml human recombinant GM-CSF (“GM-CSF”), or 3) RPMI media with 10% FBS with *Leishmania major* (MOI = 7) at the time of plating (“*Leishmania*”). The GM-CSF-treated monocytes differentiate into MDMs and were used as a positive control for all the studies. Medium was changed at day 7 and then weekly, replating any non-adherent cells into their respective wells. As illustrated in Figure 1A, a marked decline in the cell numbers was seen at day 3 after initial plating in all three conditions, though more notably in the control monocytes as compared to either GM-CSF-treated or *Leishmania*-infected monocytes. Cell numbers fell from 3×10^6^ at day 0 in all three conditions and were consistently lower in control cells as compared to either GM-CSF-treated (positive control) or *Leishmania*-infected cells at all times tested from day 3 to day 28. Control monocyte numbers declined until day 7, when their numbers stabilized through day 28. Cell numbers for GM-CSF-treated and *Leishmania*-infected groups remained significantly higher than control monocytes at all time points from day 3 to day 28 (Friedman test; p<0.05).

Next, we examined the different cell populations using light microscopy. The control cells largely retained a small, mostly rounded morphology (Figure 1B) at day 14 as compared to either GM-CSF-treated (Figure 1C; positive control) or *Leishmania*-maturated MDMs (Figure 1D). For both treatments, the monocytes were larger, more adherent and spread out with some processes, which is characteristic of mature macrophages.

Using FACS analysis, the GM-CSF-treated and *Leishmania*-maturated MDMs were larger (as assayed by forward scatter) with greater cellular complexity (assayed by side scatter) as compared to control monocytes (Figure S2). These findings were further confirmed and quantitated by FACS analysis of cell surface CD14 expression from six independent donors. This demonstrated a decreased cell surface expression of CD14 (CD14^low^) in both day 14 GM-CSF-treated and *Leishmania*-infected MDMs as compared to control monocytes (CD14^high^), again consistent with monocytes to macrophages maturation in the GM-CSF and *Leishmania*-infected cultures (Figure S2). FACS analysis for both Annexin V and propidium iodide also showed pronounced reduction in cell death for the *Leishmania*-infected monocytes compared to uninfected controls (Figure S3) Collectively, these data suggest that *Leishmania* infection of monocytes leads to less cell death and increased cellular maturation towards a macrophage phenotype compared to control monocytes.

### The effect of Leishmania infection on human monocyte proliferative capacity

**Figure 2:**
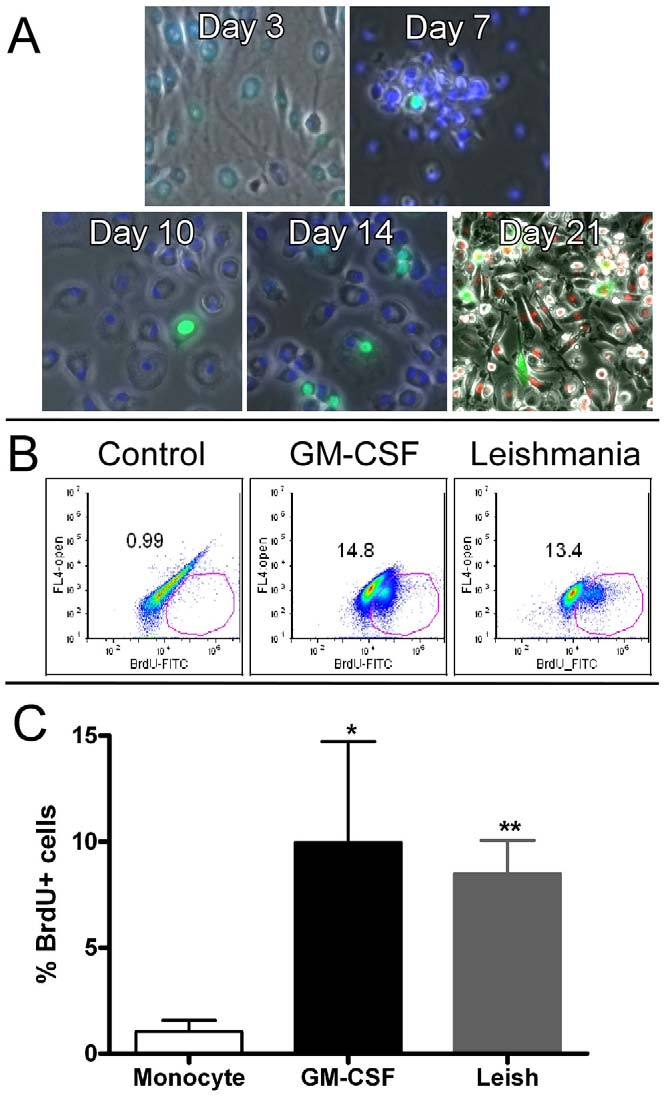
BrdU analysis. A) Monocytes were infected with *Leishmania* at day 0. BrdU reagent was added 48 hours before fixing, processing and capturing the images. B) BrdU incorporation as monitored by FACS analysis. At day 13 of maturation, the cell populations from different groups were harvested and BrdU incorporation was determined. One of six independent donors is displayed for each group. C) The six independent donors were graphed and displayed as mean and SEM for BrdU+ cells. The GM-CSF-treated and *Leishmania*-infected MDMs have significantly higher BrdU incorporation as compared to control monocytes (* = p<0.05; ** = p<0.01).

While performing the kinetic studies of *Leishmania*-infected monocytes, we observed clusters of small cells lying on top of larger, more differentiated appearing macrophages in both the *Leishmania*-infected and GM-CSF-treated (positive control) cultures but not for the control cell culture. Although, as noted above, human monocytes are generally believed not to proliferate once released from the bone marrow [18], [19], it has been more recently recognized that these cells possess far greater heterogeneity than originally believed and subpopulations of monocytes have been recently described that can re-enter the cell cycle in response to M-CSF and GM-CSF [20]–[22]. Proliferation of these monocyte subpopulations has been demonstrated by multiple techniques including uptake of BrdU and CFSE labeling [23], [24]. Thus, we next asked whether their presence might also be induced in the setting of *Leishmania* infection. To address this, we did a time-course analysis at days 3, 7, 10, and 14, examining BrdU uptake at 48 hours after treatment for the *Leishmania*-infected groups [24]. As expected, we detected a few cells that were uniformly BrdU+ (green) and nuclei counterstained with DAPI (blue) (Figure 2A). We detected a progressive increase in the numbers of BrdU+ cells over time, with maximal numbers of BrdU+ cells observed at day 14 of cell culture. Lastly, we co-labeled primary human monocytes with PKH-labeled *L. major* (orange) and then pulsed with BrdU (Figure 2A, bottom right panel Day 21). BrdU+ nuclei were seen in *Leishmania*-infected cells suggesting that infection may promote re-entry into the cell cycle for a sub-population of cells. This may be of importance to the dissemination of *Leishmania* within a host because macrophages are generally considered terminally differentiated, non-dividing cells [19].

We subsequently performed quantitative FACS analyses to compare the percentages of BrdU+ cells. As shown in a representative FACS plot, Figure 2B, a relatively large sub-population of BrdU+ cells was seen in both *Leishmania*-infected (13.4%) and GM-CSF-treated cells (14.8%) but not in control cells (<1.0%). Figure 2C summarizes results for 48 hour BrdU incorporation for seven independent donors between days 12–14. *Leishmania*-maturated MDMs demonstrated highly statistically significant (p<0.01) elevations of the percentage of BrdU+ cells as compared to control cells while GM-CSF-maturated MDMs were significantly (p<0.05) higher. We also CSFE-labeled fresh monocytes and found at least one cell division in a small subpopulation of cells for the GM-CSF-treated and *Leishmania*-infected groups (data not shown). Collectively our results are consistent with previous studies of a proliferative monocyte sub-population that can be stimulated to enter cell division by the related monokine M-CSF [21], [24]. However, of greater relevance is the demonstration that *L. major* infection of monocytes can induce an S phase environment as assayed here by BrdU incorporation. Whether this promotes cell division *in vivo*, allowing for greater dissemination of *Leishmania*, remains unclear.

### The impact of Leishmania infection on human monocyte intracellular dNTP concentration

**Figure 3:**
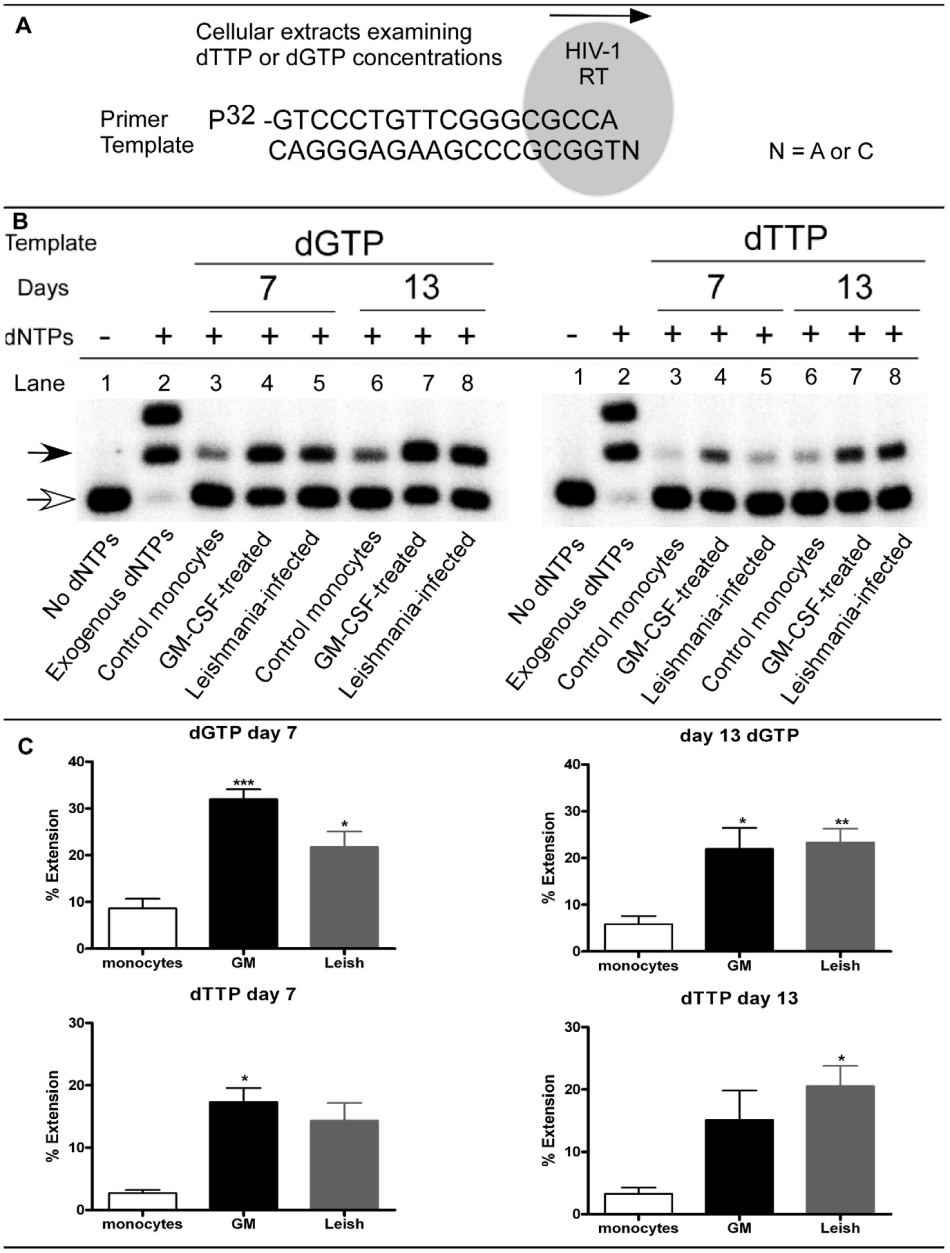
HIV-1 RT based dNTP assay. A) Diagram shows how a single nucleotide extension assay is done. The reaction contains the template, 5′ ^32^P-end-labeled primer, HIV-1 RT and cellular dNTP extract. After the reactions are completed, they are resolved on a polyacrylamide gel to determine product formation. The concentrations of dGTP and dTTP in the cellular extract will determine the amount of primer extension. B) For the different groups, primer extension products are shown for day 7 and day 13 cellular extracts. Control monocytes, GM-CSF-treated and *Leishmania*-infected cells are shown. The GM-CSF-treated cells are used as a positive control. Primer only (unextended) is indicated with an open arrow, whereas extended product formation is indicated with a filled arrow. The negative controls contain no dNTPs and are shown in lane 1. Positive controls (lanes 2) contained 50 µM of exogenous dNTP mix. Lanes 3–8 are cellular extracts from the different treatment groups. C) Graphs plotting the percent extension of dGTP and dTTP. From the primer extension assays, data was plotted for days 7 and 13 for dGTP (n = 9; top graphs) and for dTTP (n = 6; bottom graphs). Significantly different groups are displayed as * = p<0.05, ** = p<0.01 and *** = p<0.001 for the different groups as compared to control monocytes.

We employed the highly sensitive HIV-1 RT based assay for measuring cellular dNTP content [32], [42]–[45]. As depicted in Figure 3A, HIV-1 RT is bound to a template/primer complex. HIV-1 RT can extend the primer by one nucleotide, depending on the template nucleotide (N) present at the 5′ end of the template. This assay allows for the determination of differences between cellular extracts for a specific cellular dNTP. Using this assay, we compared the cellular content of dGTP (purine) and dTTP (pyrimidine) for the different treatment groups. Figure 3B shows a representative result for primer extension of dGTP (left panel) and dTTP (right panel). Summary results for nine individual donors are presented in graph form in Figure 3C and are summarized below.

In Figure 3B, left side panel, dGTP levels were assayed at days 7 and 13, while the right side panel shows dTTP analysis for the same days. In lanes 1 for both dGTP and dTTP analysis, no dNTPs were added to the reaction, leading to no extension product of the labeled primer (open arrow). In lanes 2, exogenous dNTPs were added as a positive control to show extension of all primers in the reactions (closed arrow). In lanes 3–8, days 7 and 13 cellular extracts were analyzed. Content of dGTP were notably higher in GM-CSF- and *Leishmania*-maturated MDMs as compared to untreated control cells at day 7 (lanes 4 and 5) and day 13 (lanes 7 and 8) after treatment. In comparison, dTTP concentrations at day 7 were slightly higher for the GM-CSF-maturated MDMs (lanes 4, positive control) as compared to the control and *Leishmania*-maturated MDMs. At day 13, we detected much higher dTTP concentrations in the GM-CSF and *Leishmania*-maturated MDMs at day 13 (lanes 7 and 8) as compared to the control group (lanes 6). These data demonstrate that *Leishmania* infection can lead to notable increases in cellular dNTP concentrations and this conclusion is fully validated by quantification of the assay results in nine individual donors (Figure 3C). Results for dGTP (Figure 3C, upper panels) demonstrated statistically significant increases for GM-CSF-matured and *Leishmania*-infected MDMs as compared to control monocytes at day 7; by day 13 dGTP increases were now highly significantly elevated in *Leishmania* and still significantly elevated in the GM-CSF-maturated MDM groups as compared to controls. The results for dTTP at day 7 (Figure 3C, lower left panel) trended higher in *Leishmania*-maturated MDMs as compared to monocyte controls but only reached significance in GM-CSF-maturated MDMs. However at day 13, (Figure 3C, lower right panel) *Leishmania*-maturated MDMs were significantly increased in dTTP concentrations as compared to monocyte controls. These data demonstrate that *Leishmania* infection of monocytes induces elevation of both purine and pyrimidine concentrations in the host cell. The finding of elevated purine levels is particularly intriguing in light of the fact that *Leishmania* species are entirely dependent on host cell synthesis for their supply of purine nucleotides [46].

### Exposure of primary human monocytes to Leishmania induces elevated levels of RNR subunits

**Figure 4:**
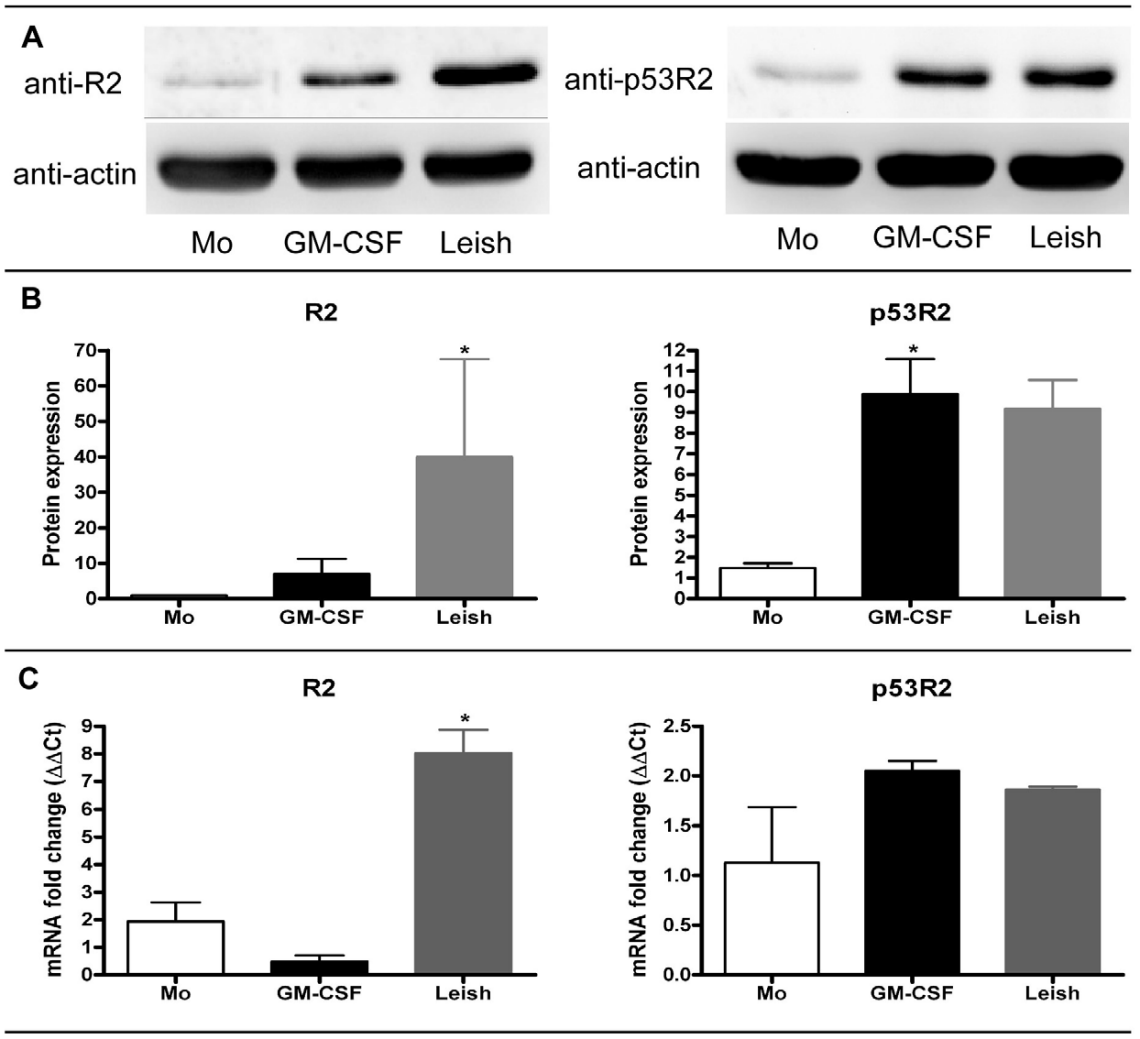
Western blot analysis of ribonucleotide reductase. A) Cellular lysates from different treatment groups were analyzed for ribonucleotide reductase R2 and p53R2 subunits. Afterwards, blots were stripped and re-probed for actin. Freshly isolated monocytes (Mo), day 13 maturated GM-CSF (GM-CSF) and *Leishmania* (Leish) MDMs are shown. B) Quantitiation of western blots was done. Freshly isolated monocytes were set to 1 and increases in R2 and p53R2 expression levels for GM-CSF- and *Leishmania*-maturated MDMs groups are shown. Mean and SEM are displayed for four independent donors. C) qRT-PCR analysis was done on total cellular RNA extracts. mRNA fold changes for the different treatment groups (n = 3) are graphed as mean and SEM. Significantly different groups (p<0.05) as compared to monocyte control group are indicated with an asterisk (*).

Mammalian RNR is a dimeric enzyme essential for catalyzing the direct reduction of relatively large intracellular pools of ribonucleotides into the corresponding deoxyribonucleotides for DNA synthesis. The catalytic enzyme is a heterodimer, containing two subunits of R1 and either two subunits of R2 or p53R2. Expression of the R2 subunit is strictly limited to the S phase of the cell cycle [47]. As shown in Figure 4A, western blot analyses were done for R2 and p53R2 on cell extracts using freshly isolated monocytes, day 13 GM-CSF or *Leishmania*-maturated MDMs. As shown in Figure 4B, we quantitated the western blots for four independent donors and found that R2 was significantly (p<0.05) increased in the *Leishmania*-maturated MDMs over control monocytes. For the p53R2, we found a significant increase in the GM-CSF-treated cells but the increase failed to reach significance for the *Leishmania*-infected cells when compared to monocytes, which were set to 1. Moreover, the R2 and p53R2 antibodies were specific for human ribonucleotide reductase and did not cross-react with *L. major* (data not shown). Collectively, these data show that 1) R2 subunit expression, which is S phase linked, is significantly increased upon *Leishmania* infection, and 2) that infection indirectly leads to an increase in the p53R2 subunit, which is involved in increasing cellular dNTP concentrations in non-dividing cells.

### Analysis of Ribonucleotide reductase R2 and p53R2 transcription

Next, quantitative reverse transcriptase quantitative PCR (qRT-PCR) using Taqman analysis was performed in three individual donors to examine whether the observed increase in RNR R2 subunit and P53R2 protein expression showed transcriptional regulation (Figure 4C). Consistent with the significantly increased protein expression of the RNR R2 subunit seen by western blot, significantly increased transcription was seen in *Leishmania*-infected monocytes as compared to GM-CSF-treated MDM and control monocytes. It is also possible that these results may be due, at least in part, to an increase in RNR R2 transcript stability. In contrast, increased expression of p53R2 protein likely occurs due to post-transcriptional regulation as no significant elevation of transcription was seen in either the GM-CSF-treated or *Leishmania*-infected MDMs as compared to control monocytes.

### Results of HIV-1 D3 GFP transduction for the different treatment groups

**Figure 5:**
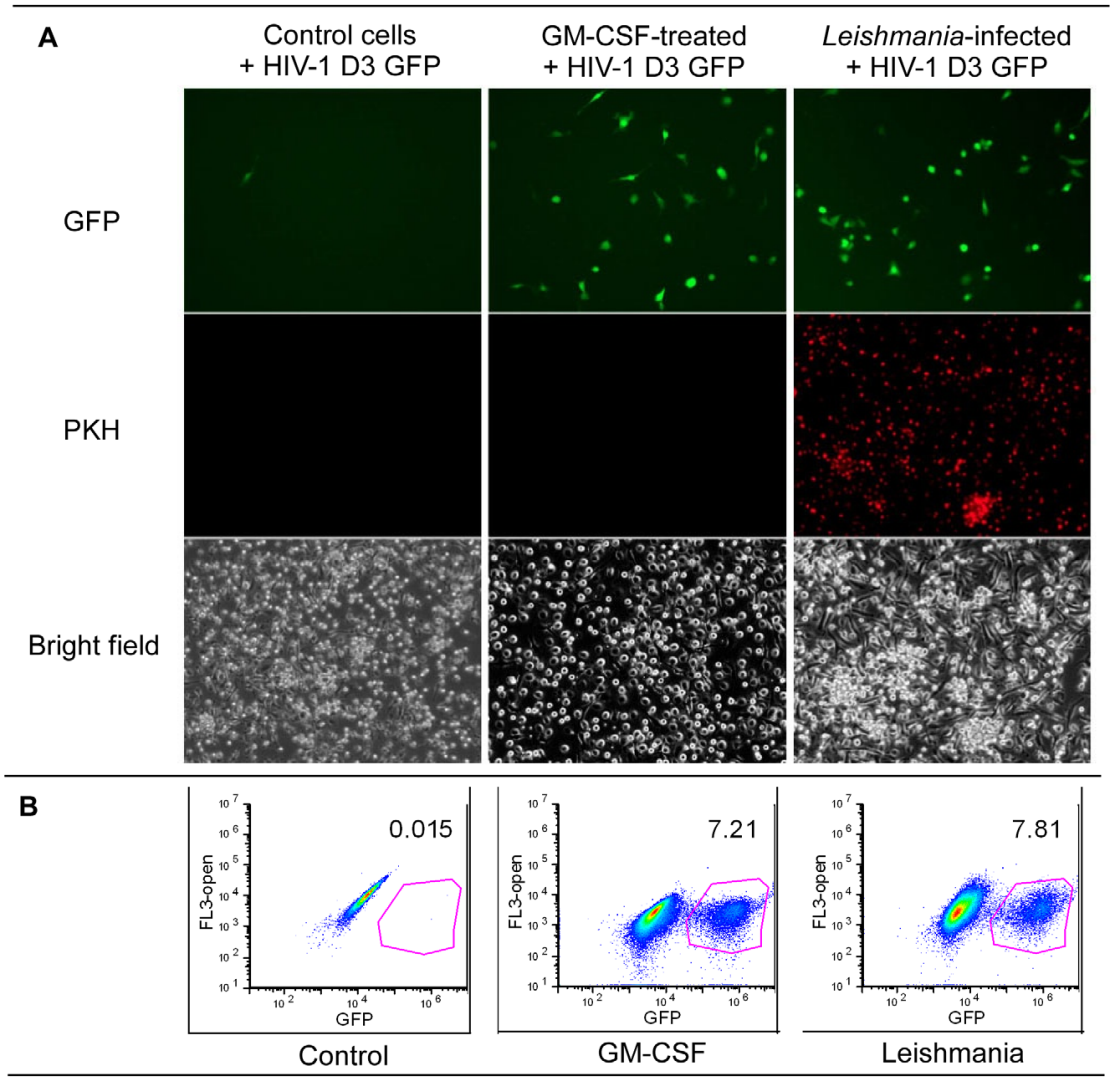
HIV-1 D3 vector analysis. A) Control, GM-CSF-treated and *Leishmania*-infected cells were transduced with HIV-1 D3 GFP vector. Twenty-four hours after transduction, the cells were examined for GFP expression (top row). Very few monocytes were GFP+, whereas more cells were GFP+ for the GM-CSF-treated and *Leishmania*-infected groups. For this experiment, the *L. major* were labeled with PKH dye and showed that the monocytes were infected. Bright field images were captured for the different groups. B) FACS analysis was done of three different cell populations. Data is representative of four different donors done at 24 h. Complete data sets for 24 h and 48 h are shown in Table 1.

As noted above, cellular dNTP levels serve as a biomarker for the replicative capacity of mammalian cells, a finding corroborated by the presence of consistently higher dNTP levels in dividing cells as compared to non-dividing cells [25]–[30]. HIV-1 replication efficiency is also directly correlated with the cellular dNTP concentration, and we and others have reported that it proceeds with far greater efficiency in tumor cells or PHA-stimulated CD4+ T cells in which the average dNTP level is 150–225 times higher than in non-dividing monocytes/macrophages [32], [33]. Given our findings that *Leishmania* infection induces both significant elevation of dNTP levels and replication capacity in MDMs, we examined whether transduction of *Leishmania*-maturated MDMs with a VSV-g pseudotyped HIV-1 vector, designated HIV-1 D3 GFP, resulted in accelerated HIV-1 expression, as determined by GFP expression. Six days after isolation, control cells, GM-CSF maturated MDMs, and PKH-labeled (red) *Leishmania*-maturated MDMs were transduced in 6-well dishes with equal amounts of HIV-1 D3 GFP vector. We examined cells by bright field and fluorescence microscopy 24 hours later (Figure 5A). HIV-1 D3 GFP expression (“GFP” [green-top 3 panels]) was markedly enhanced, relative to control cells, in both the GM-CSF- and *Leishmania*-maturated MDMs (upper middle and right-sided panels, respectively), consistent with both strikingly increased intensity and numbers of HIV-1 D3 GFP transduced cells in these two conditions relative to control cells (Figure 5A; top left panel). Only a rare control cell appeared to express HIV-1 D3 GFP. GM-CSF- and *Leishmania*-maturated MDMs had many more cells expressing GFP as compared to control cells. *Leishmania* maturated MDMs labeled with PKH showed comparable numbers of GFP+ cells/field as compared to GM-CSF-maturated MDMs (Figure 5A; middle and top right panels). We next quantified the three different groups by FACS analysis (Figure 5B and Table 1). For these studies, the *Leishmania* were not labeled with PKH dye. As shown in Table 1, cells from four independent donors were examined at 24 and 48 h after the addition of the HIV-1 D3 GFP vector. The percent of GFP+ cells for *Leishmania*-maturated MDMs were consistently higher as compared to the control cell group with a somewhat weaker trend to higher percentage of GFP+ cells also found in the GM-CSF-maturated MDMs as compared to control cells.

**Table 1:** Percent HIV-1 D3 GFP Transduction.

	% GFP+ cells (24 h)		
Donor	Control	GM-CSF	Leishmania
1	0.02	7.21	7.81
2	0.03	5.13	3.59
3	0.10	0.59	1.79
4	0.16	0.62	3.42

	% GFP+ cells (48 h)		
Donor	Control	GM-CSF	Leishmania
1	0.02	0.25	1.31
2	0.07	3.08	6.26
3	1.84	4.79	12.4
4	2.14	7.69	6.27

### Real Time PCR analysis for 2LTR circles

**Figure 6:**
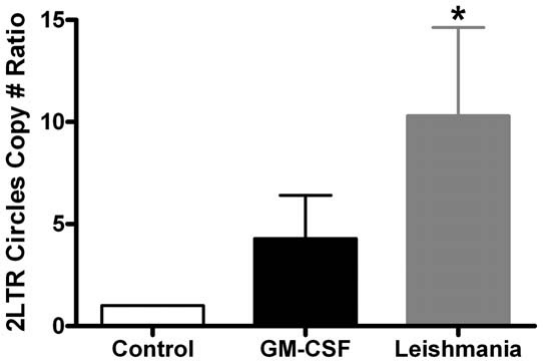
2LTR circle copy number ratio. The different treatment groups were treated with the D3 GFP vector. At 48 h after transduction, total cellular DNA was collected and analyzed by real-time PCR. The control group for each donor was set to 1 and then compared to GM-CSF- and *Leishmania*-maturated MDMs groups. Mean and SEM are plotted. The significantly different group (p<0.05), as compared to control, is indicated with an asterisk. Seven independent donors were analyzed.

Next, we examined 2LTR circles, an indicator for the completion of DNA synthesis by HIV-1 reverse transcriptase but a failure of the DNA to integrate into the host genome. As shown in Figure 6, the 2LTR circles copy number ratio was significantly higher (*, *p*<0.05) in the *Leishmania* maturated MDMs group as compared to control cell group (set to 1.0). The 2LTR circle number ratio for GM-CSF-maturated MDMs group is higher than the controls cells, but did not achieve statistical significance. Collectively, these data indicate that *Leishmania* infection promotes a pro-HIV-1 environment within the cell, leading to higher dNTP concentrations that allow for more efficient viral infection.

## Discussion

In mutually endemic areas of the world, *Leishmania* species and HIV-1 primarily co-infect mononuclear phagocytes of infected mammalian hosts. It is widely believed that *Leishmania* infection found concurrently with HIV-1 induces a state of chronic immune activation leading to subsequent increased HIV-1 viral load and accelerated progression to AIDS [48]. Although the mechanisms underlying this phenomenon are incompletely understood, *in vitro* studies to date have implicated a variety of *Leishmania*-induced pro-inflammatory cytokines including TNF-α, IL-1, and IL-6, in stimulating HIV-1 replication in both monocytoid cell lines and macrophages [17], [49]–[52]. For example, the induction of TNF-α is known to activate HIV-1 replication through mechanisms involving transcriptional activation of nuclear factors binding to NF-κB sequences in the HIV-1 LTR [49], while IL-6 and IL-1 appear to promote HIV-1 replication through less well-defined NF-κB-independent transcriptional and post-transcriptional mechanisms [51], [52]. In this study, a novel mechanism is described in which *Leishmania* infection of HIV-1 infected CD14+ primary human monocytes promotes accelerated HIV-1 expression by induction of MDMs RNR with subsequent elevation of intracellular dNTP concentrations.

This same mechanism could explain numerous previous *in vitro* and *in vivo* observations of accelerated HIV-1 replication in AIDS clinical trials for patients treated with GM-CSF [53]. Soon after the recognition that HIV-1 was the etiologic agent of AIDS, it was recognized that physiological stimuli, including GM-CSF, could exert an inductive effect on HIV-1 replication in infected monocytoid cells, though the potential mechanisms for this induction have remained unknown [54]. Most subsequent studies have largely confirmed this original observation [55]–[61], although some others have demonstrated opposite results with the suppression of HIV-1 replication [62], [63]. *In vivo*, however, the results of four clinical trials using GM-CSF therapy in HIV-1 infected patients not treated with anti-retroviral drugs all demonstrated increased plasma levels of HIV-1 RNA and p24 antigen as compared to control patients [64]–[67]. Most recently, the results of the previous negative *in vitro* studies, in which treatment with GM-CSF may have lowered HIV-1 replication, may be reconciled: the majority of results showed that up-regulation of viral replication was generally enhanced in GM-CSF-maturated MDMs when grown at low densities, whereas more crowded cultures of MDMs and excessive acidification of the medium led to suppressed viral replication [68].

Although GM-CSF treatment promotes maturation of monocytes into macrophages, which are terminally differentiated, non-dividing cells, there is an emerging awareness that, although human monocytes do not proliferate in the steady state, a proliferative monocyte sub-population exists that can re-enter the cell cycle in response to both GM-CSF and M-CSF [18], [19], [21], [24], [69]. *Leishmania*-infected monocytes/macrophages have been found to be able to produce a variety of colony stimulating factors, most notably GM-CSF [70]–[72]. Here we confirm that monocyte sub-populations treated with GM-CSF are able to re-enter the cell cycle and show, for the first time, that *Leishmania* infection promotes an S-phase environment in normally quiescent monocyte sub-populations. Statistically significant elevated percentages of BrdU+ cells were found in *Leishmania*-infected MDMs compared to uninfected controls (Figure 2C). Further, both monocyte maturation and proliferation occurred through a mechanism independent of GM-CSF as treatment with a high concentration of neutralizing antibody, fully sufficient to block the effects of 5 ng/ml added GM-CSF had no effect on the *Leishmania*-infected cells (Figure S4). These findings are in accord with newly described rodent data demonstrating local *in situ* proliferation of tissue macrophages in response to infection with a rodent filarial nematode [73] and a previous study demonstrating *in situ* proliferation of macrophages in the lungs of hookworm-infected mice [74].

The promotion of monocyte proliferation by both GM-CSF treatment and *Leishmania* infection has profound implications for monocyte cell biology. We now report the quite novel finding that monocyte proliferation, induced by the presence of GM-CSF and, more potently by infection with *L. major*, also promotes significantly higher dNTP levels at days 7 and 13 in culture as compared to freshly isolated peripheral blood monocytes. Elevated synthesis of the purine, dGTP in particular, was highly statistically significant in day 13 *Leishmania*-infected MDMs compared to levels in control monocytes (Figure 3C). In addition, induction of cellular RNR, the enzyme catalyzing the direct reduction of ribonucleotides to their corresponding dNTPs was found to be significantly elevated in the *Leishmania*-maturated MDMs. Specifically, an approximately 40-fold increase in RNR protein levels was observed in immunoblots of day 13 *Leishmania*-maturated MDMs versus freshly isolated monocytes using an antibody directed against the R2 subunit of human RNR (Figure 4B). That this induction of RNR R2 is regulated at the transcriptional level is supported by the similarly statistically significant elevation of RNR R2 RNA assayed by qRT-PCR (Figure 4C). These findings are particularly intriguing in that the expression of the R2 subunit is known to be strictly and specifically restricted to the S phase of the cell cycle [47], consistent with the observed induction of cell cycle re-entry in both *Leishmania-* and GM-CSF-maturated MDMs.

The present demonstration that *Leishmania* infection of human monocytes induces elevated dNTP concentrations also has far-reaching implications for *Leishmania* pathogenesis. Unlike their mammalian hosts, *Leishmania* lack the metabolic machinery needed for purine nucleotide synthesis. They must therefore rely on the host cell production of purines and have evolved an obligatory purine salvage pathway for this purpose [46]. The dimeric enzyme ribonucleotide reductase is the major source of dNTPs in mammalian and other cells, forming them from the far more abundant pool of rNTPs by the removal of the 2′ OH on the ribose sugar moiety [75]. Our finding that *Leishmania* infection of human monocytes induces MDMs upregulation of RNR (Figure 4B) is fully consistent with the elevated dNTP concentrations noted above and represents an elegant evolutionary adaptation by which *Leishmania* can salvage necessary host purines (and pyrimidines). A more recent consequence of *Leishmania*-mediated induction of host RNR and elevated dNTP concentrations has been to provide a highly permissive environment for HIV-1 replication in the setting of co-infection. These findings are especially significant in light of data that HIV-1 proviral DNA synthesis in non-dividing cells is slower than in dividing cells [76], and can be accelerated by experimentally elevating the intracellular dNTP concentration [42]. They may also be of particular relevance in the setting of infection with *Leishmania*, in which rapid proliferative expansion of local splenic and bone marrow monocyte/macrophage progenitor populations has been described [70]. In this setting, elevated dNTP concentration would also be expected with accompanying enhancement of HIV-1 replication in such dividing cells.

HIV D3 GFP transduction, a model for HIV-1 infection, is also markedly enhanced in these matured cells (Figures 5A, 5B, and Table 1). Both the fluorescent microscopic and flow cytometry results demonstrated substantially increased numbers of HIV-1 D3 GFP+ transduced cells in the setting of *Leishmania* infection. These findings were further confirmed by a statistically significant elevation of the 2LTR circle copy number ratio in *Leishmania* infected MDM compared to control monocytes by qPCR (Figure 6).

Our results for MDMs maturated by GM-CSF treatment or infection with *Leishmania* conform well to the majority of studies showing enhanced HIV-1 replication, most likely due to monocytes maturating into macrophages. This is a critical finding in that we have recently reported that HIV replication efficiencies in a wide variety of relevant cell types, including monocytes and macrophages, is directly related to the relative intra-cellular dNTP concentrations [31], [32]. Thus, the finding of elevated dNTP levels in both GM-CSF- and *Leishmania*-maturated human MDMs, as compared to both freshly isolated monocytes and untreated control cells, offers a novel mechanism to explain both the present results as well as prior *in vitro* and *in vivo* studies that demonstrate accelerated HIV-1 replication in both GM-CSF-treated and *Leishmania* co-infected patients [55], [67], [77], [78]. These results are consistent with the 200–1500 times decrease in replication competence of wild-type HIV-1 in monocytes as compared to the corresponding differentiated MDMs [33].

The present study represents the first demonstration that *Leishmania* promotes both maturation and proliferation phenotypes in primary human monocytes. During this process we detected elevated intracellular dNTP pools in *Leishmania*-infected cells, which allows more efficient replication of intracellular co-infected HIV-1. This observation of enhanced pathogen expression in co-infected target cells may be a more generalized phenomenon. For example, the course of HIV-1 related immunodeficiency is also known to be accelerated by active infection with *Mycobacterium tuberculosis* (MTB) [79], and *in vitro* studies have demonstrated that MTB-infection of MDMs subsequently infected with HIV-1 produce increased levels of virus as compared to MDMs uninfected with MTB [80]. In matched CD4+ T cell cohorts, both HIV-1 viral load and heterogeneity are increased by MTB infection. In addition, infection of monocytes/macrophages with two other clinically relevant *Mycobacterium* was found to enhance HIV-1 replication both *in vitro* and *in situ* [81]–[83]. Conversely, patients co-infected with HIV-1 and MTB have altered granulomas within the lung [84]. Also higher bacterial burden was detected for HIV-1 and MTB co-infection of MDMs *in vitro* [85]. Our data suggests that we are just beginning to understand the synergy between virus and parasite co-infections of human cells.

## Materials and Methods

### Ethics statement

These experiments used primary human primary monocytes obtained from human buffy coats (New York Blood Services, Long Island, NY). These are pre-existing materials that are publicly available, and there is no subject-identifying information associated with the cells. As such, the use of these samples does not represent human subjects research because: 1) materials were not collected specifically for this study, and 2) we are not able to identify the subjects.

### Cells

Primary human monocytes were isolated from the peripheral blood buffy coats by positive selection using MACS CD14+ beads as previously described [32]. Three culture condition were used: 1) RPMI 1640 containing 10% FCS and Penicillin/Streptomycin antibiotics without further supplements indicating “control” monocytes, 2) RPMI containing 10% FCS, Pen/Strep antibiotics and 5 ng/ml human recombinant GM-CSF (R&D Systems) indicating “GM-CSF-treated” monocytes, or 3) RPMI 1640 containing 10% FCS, Penicillin/Streptomycin antibiotics and *Leishmania major* (MOI = 7) indicating “*Leishmania*-infected” monocytes. *Leishmania major* promastigotes (strain WHOM/IR/–/173) were grown to stationary phase culture and infectious metacyclic promastigotes were isolated by negative selection using peanut agglutinin [86]. *L. major* were labeled with 2 µM PKH26 fluorescent cell dye (Sigma) as per manufacturer's protocol.

*HIV-1 D3 GFP vector generation:* HIV-1 D3 GFP vector encodes the HIV-1 NL4-3 genome with the eGFP gene in place of the HIV-1 nef gene and has a deleted envelope [32]. To generate virus, 293T cells in T225 flasks were transfected with 60 µg pD3-HIV and 10 µg pVSV-g plasmids using 140 µl polyethyenimine (1 mg/ml) in 37 ml DMEM media/flask. At day 1 of HIV-1 production, media was discarded and replaced with fresh DMEM media. At day 2, media was harvested and replaced with fresh DMEM media. The media was centrifuged at 2500 RPM for 7 minutes to remove cellular debris, and then stored at 4°C in T75 flask. Day 3 media was harvested and processed as described for day 2. HIV-1 D3 GFP was concentrated using ultracentrifugation (22K RPM for 2 h in a SW28 rotor). Viral pellets were DNase I digested for 1 h at 37°C. Afterwards, debris was removed by centrifugation (14K for 5 minutes). Sample aliquots were frozen at −80°C until used. Different groups were transduced with HIV-1 D3 GFP and then the samples were analyzed using Accuri C6 flow cytometer monitoring GFP expression at 24 h or 48 h after transduction. Data files were analyzed using FlowJo software (TreeStar).

### Primer extension assay

Nucleotide incorporation assay employs a 19-mer DNA template (3′-CAGGGAGAAGCCCGCGGTN-5′). The N indicates the change in template for detecting a specific dNTP within the cellular extract. The template is annealed to a 5′ end ^32^P-labeled 18-mer DNA primer (5′-GTCCCTGTTCGGGCGCCA-3′). HIV-1 RT is used for this reaction [87]. 1×10^6^ cells for control monocytes, GM-CSF-treated MDMs, and *Leishmania*-infected MDMs were collected and lysed with 60% cold methanol. Cellular debris was cleared by 14K centrifugation. Supernatant was dried. Pellet was resuspended in 20 µl reaction buffer (50 mM Tris-HCl, pH 8 and 10 mM MgCl_2_). Two microliters were used in the primer extension assay.

### BrdU labeling

Forty-eight hours before harvesting, cells were pulsed with 300 µM BrdU. For microscope analysis, media was removed and the 6-well plate was washed once with PBS. Cells were fixed for using 4% paraformaldehyde for 20 minutes and then washed with PBS. Two milliliters of Target Retrieval Solution (Dako) was added and plates were heated in a rice cooker for 15 minutes at 95°C. Afterwards the plates were removed and allowed to cool. Cells were stained with rat anti-BrdU-FITC antibody (AbD Serotec) for 20 minutes at 4°C. Images were captured using a Zeiss microscope. For FACS analysis, on the day of harvest, the free cells were collected while the adherent cells were Trypsin treated for 30 minutes before scraping the 6-well plate. Both free and adherent cell populations were pooled, centrifuged at 1200 RPM for 5 minutes. Supernatant was removed and the cells were fixed using 4% paraformaldehyde for 20 minutes. After fixing, the cells were washed once with PBS. The cells were stored at 4°C until processing for BrdU staining. For BrdU staining, cells were transferred to a 6-well plate containing 2 ml of Target Retrieval Solution and heated in a rice cooker for 15 minutes at 95°C. Afterwards the plates were removed and allowed to cool. Cells were transferred to tubes and cells washed once with PBS. Next the cells were stained with rat anti-BrdU-FITC antibody for 20 minutes at 4°C. The sample data were collected using an Accuri C6 flow cytometer.

### Western blot analysis

Samples were processed in RIPA buffer containing 1 µM DTT, 10 µM PMSF, 10 µl/ml phosphatase inhibitor (Sigma) and 10 µl/ml protease inhibitor (Sigma). The cells were sonicated with 3X, 5 second pulses, to ensure complete lysis. Cellular debris was removed by 15K RPM centrifugation for 10 minutes. Supernatants were stored at −80°C before use. Cell lysates (25 µg) were resolved on an 8% SDS-PAGE gel. Proteins were transferred to a nitrocellulose membrane. The membrane was blocked with 2% non-fat milk in TBST for 1 h, followed by the addition of primary goat anti-R2 antibody (Santa Cruz Biotechnology) and incubation overnight at 4°C. The next day, the membrane was washed (3X, 20 minutes with TBST) followed by staining with donkey anti-goat HRP for 1 h at room temperature. The membrane was washed 3× with TBST and developed using SuperSignal West Femto Kit (Thermo Scientific). The immunoblot was then stripped and re-probed for actin. Images were captured using a BioRad ChemiDoc Imager.

### Analysis of Ribonucleotide reductase R2 and p53R2 mRNAs by quantitative reverse transcriptase PCR

4×10^6^ cells were lysed and RNA prepared using the RNeasy Mini Protocol as per the manufacturers' instructions (Qiagen, Valencia, CA). Pre-mixed Taqman primer/probe sets for RNR R2 and p53R2 were obtained from Life technologies (Cat numbers Hs01072069_gi and Hs00968432_m1, respectively). Template RNA was diluted to 80 ng/µl and 4 µl from each sample, mixed with Express One-Step SuperScript qRT-PCR reagents, was ran in triplicate using an Applied Biosystems 7300 Real Time thermocycler. Data were normalized to GAPDH mRNA.

### 2LTR circle analysis

Genomic extracts were prepared using QuickGene-810 Nucleic Acid Isolation System (FujiFilm Global). The DNA was assayed for 2LTR circles by real time PCR using the following primers: 5′-LTR region — 5′-GTGCCCGTCTGTTGTGTGACT-3′ and 3′LTR region — 5′-CTTGTCTTCTTTGGGAGTGAATTAGC-3′, and the probe 5′-6-carboxylfluorsecein-TCCACACTGACTAAAAGGGTCTGAGGGATCTCT-carboxytetramethylrhodamine-3′ (IDT). All samples were normalized to total DNA. The control samples for each donor were set to 1.0 and 2LTR circle copy number ratio was plotted.

### Graphing and statistical analysis

Prism software was used for plotting the data. All the data sets were compared for significant difference using ANOVA analysis (Friedman test).

## Supporting Information

Figure S1 **Monitoring** ***Leishmania*** **infection of monocytes.** Three independent donors were plated in 6-well plates at 1 million monocytes alone or with 7 million PKH-labeled Leishmania. At days 2 and 4 after plating, PKH+ cells were monitored using FACS analysis. Data for the different donors are plotted as mean and SEM.
(TIF)

Figure S2 **Phenotypic analysis of different cell populations.** Three independent donors were examined by FACS analysis at day 7 of maturation. FSC and SSC for the different populations, were examined for freshly isolated, floating (F) and adherent (A) cells. As clearly shown, freshly isolated monocytes have a smaller FCS and SSC as compared to the other groups; day 7 control monocytes, GM-CSF-treated and Leishmania infected cells. Next, we examined CD14 expression levels by FACS analysis having a high and low gating for mean fluorescent intensity (MFI). For the floating cells, CD14 MFI was highest for the control monocytes (Mo(F)), and was reduced for the remaining cell subsets. Finally, the percentage of CD14 low and CD14 high cells were plotted. Floating cells were CD14 high, whereas adherent cells were CD14 low.
(TIF)

Figure S3 **Determining apoptosis for control monocytes, GM-CSF maturated and** ***Leishmania*** **maturated MDMs.** Annexin V and propidium iodide (PI) staining of total cells (both adherent and non-adherent) were monitored in all experiments by FACS analysis. A) The untreated control monocytes had 33.2% apoptotic cell death as measured by annexin V and 21% necrotic cell death as assayed by propidium iodide. B) The GM-CSF-maturated MDMs, positive control, had 1.65% apoptotic cell death and roughly 2% necrotic death, and C) the *Leishmania*-maturated MDMs had roughly 2.1% apoptotic cell death and 4% necrotic cell death. Our findings suggest that infection of monocytes with *Leishmania* promoted cell survival comparable to the positive control monocytes treated with GM-CSF.
(TIF)

Figure S4 **Anti-GM-CSF treatment does not block** ***Leishmania*** **-infected maturation of monocytes.** GM-CSF-treated and *Leishmania*-infected monocyte cultures were treated with isotype control or anti-GM-CSF antibodies (10 µg/ml). Images were captured at day 5 of culture.
(TIF)
