# Supplementary figures and images for: Leishmania Induces Survival, Proliferation and Elevated Cellular dNTP Levels in Human Monocytes Promoting Acceleration of HIV Co-Infection
Source: PLoS Pathog. 2012 Apr 5;8(4):e1002635. doi: 10.1371/journal.ppat.1002635 (PMC3320607; doi:10.1371/journal.ppat.1002635)

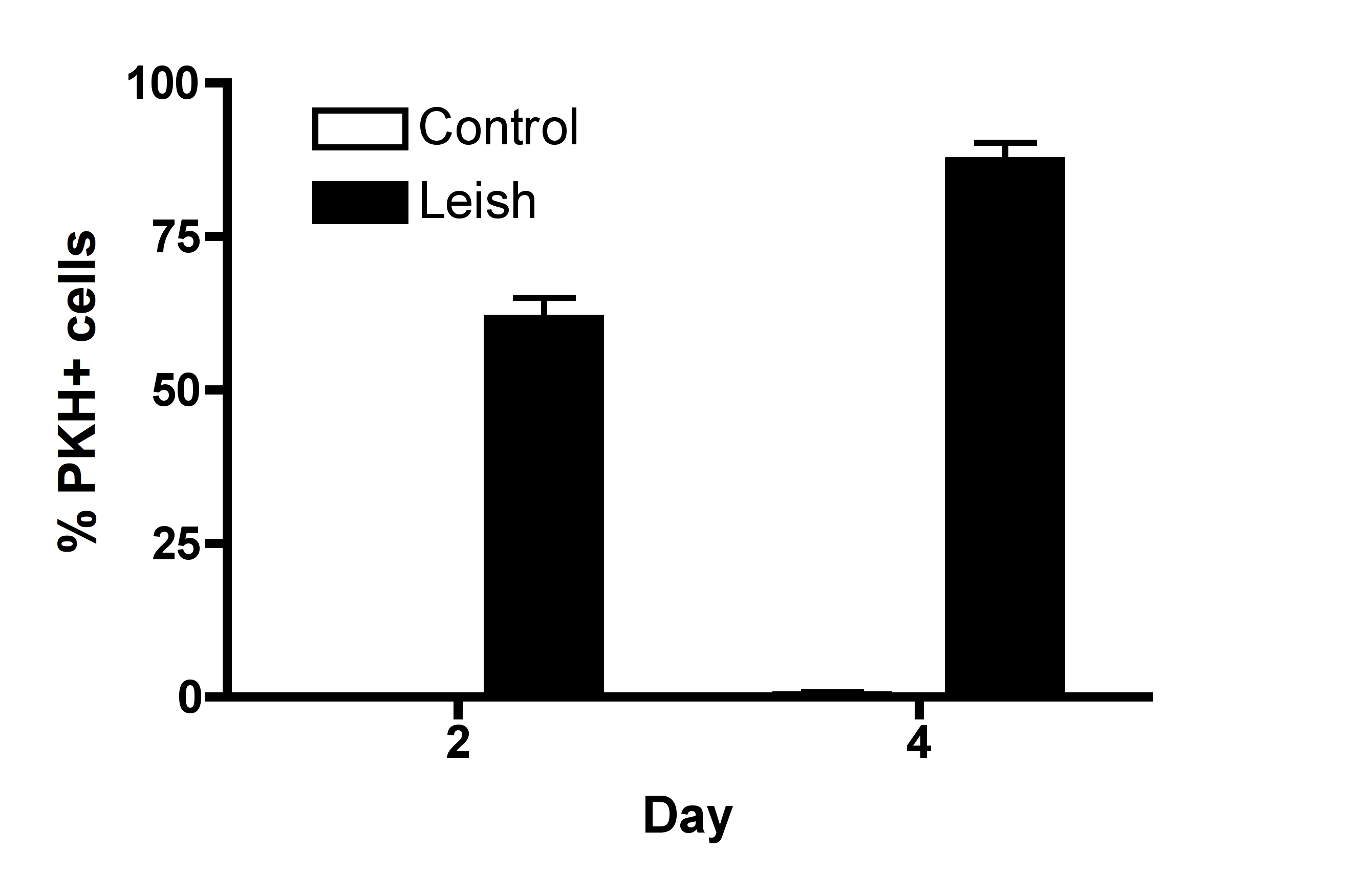

Supplement: Figure S1 — Monitoring Leishmania infection of monocytes. Three independent donors were plated in 6-well plates at 1 million monocytes alone or with 7 million PKH-labeled Leishmania. At days 2 and 4 after plating, PKH+ cells were monitored using FACS analysis. Data for the different donors are plotted as mean and SEM. (TIF) [file ppat.1002635.s001.tif]

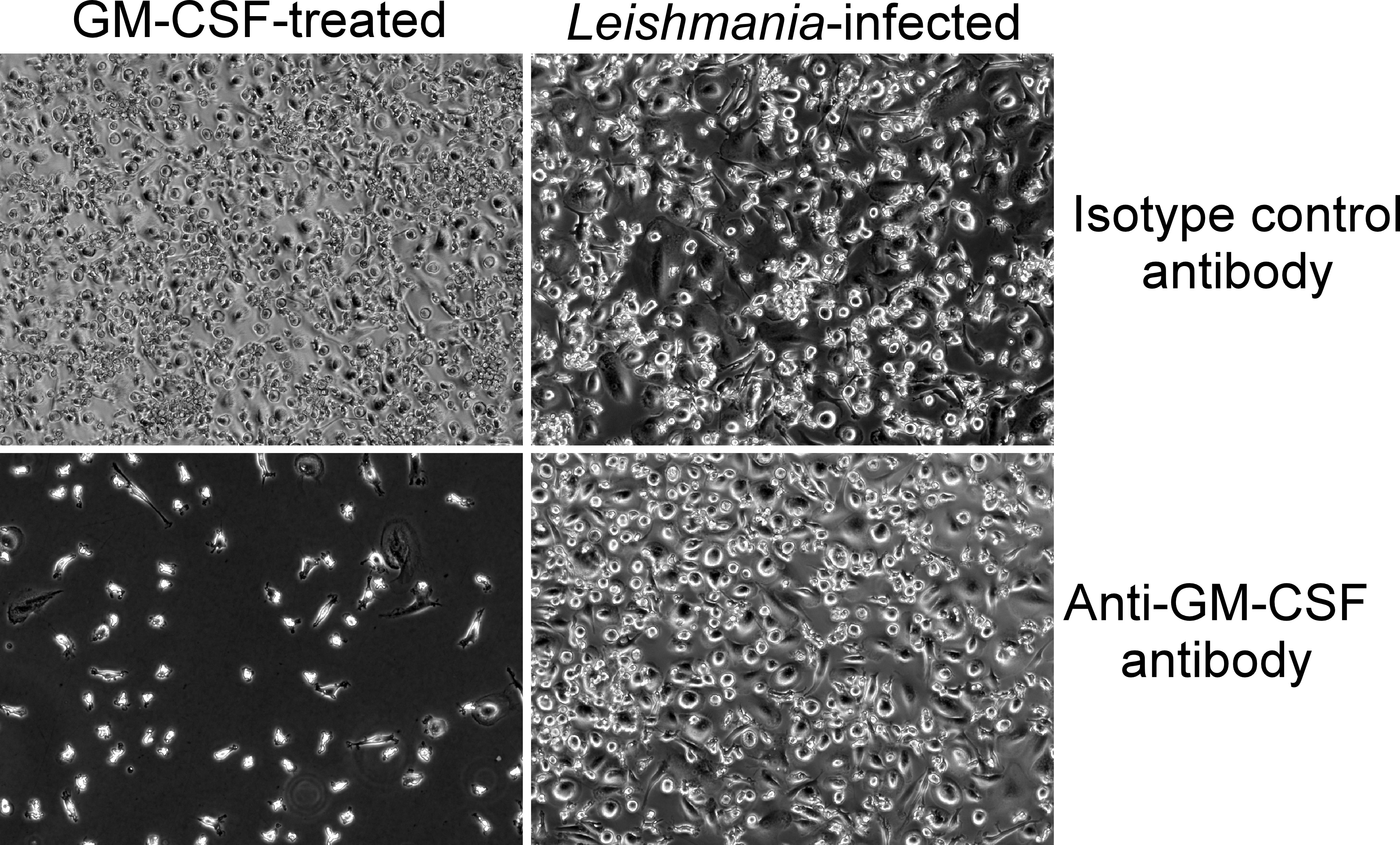

Supplement: Figure S4 — Anti-GM-CSF treatment does not block Leishmania -infected maturation of monocytes. GM-CSF-treated and Leishmania-infected monocyte cultures were treated with isotype control or anti-GM-CSF antibodies (10 µg/ml). Images were captured at day 5 of culture. (TIF) [file ppat.1002635.s004.tif]
